# Brain microbiota disruption within inflammatory demyelinating lesions in multiple sclerosis

**DOI:** 10.1038/srep37344

**Published:** 2016-11-28

**Authors:** W. G. Branton, J. Q. Lu, M. G. Surette, R. A. Holt, J. Lind, J. D. Laman, C. Power

**Affiliations:** 1 Department of Medicine, University of Alberta, Edmonton AB Canada; 2 Department of Laboratory Medicine & Pathology, University of Alberta, Edmonton AB Canada; 3 Department of Psychiatry, University of Alberta, Edmonton AB Canada; 4 Department of Medicine, McMaster University, Hamilton ON Canada; 5 Genome Sciences Centre, Vancouver BC, Canada; 6 Department of Neurosciences, Section of Medical Physiology, Faculty of Medical Sciences, University Medical Center Groningen, University of Groningen, Groningen Netherlands; 7 Multiple Sclerosis Centre, University of Alberta, Edmonton AB Canada

## Abstract

Microbial communities reside in healthy tissues but are often disrupted during disease. Bacterial genomes and proteins are detected in brains from humans, nonhuman primates, rodents and other species in the absence of neurological disease. We investigated the composition and abundance of microbiota in frozen and fixed autopsied brain samples from patients with multiple sclerosis (MS) and age- and sex-matched nonMS patients as controls, using neuropathological, molecular and bioinformatics tools. 16s rRNA sequencing revealed Proteobacteria to be the dominant phylum with restricted diversity in cerebral white matter (WM) from MS compared to nonMS patients. Both clinical groups displayed 1,200–1,400 bacterial genomes/cm^3^ and low bacterial rRNA:rDNA ratios in WM. RNAseq analyses showed a predominance of Proteobacteria in progressive MS patients’ WM, associated with increased inflammatory gene expression, relative to a broader range of bacterial phyla in relapsing-remitting MS patients’ WM. Although bacterial peptidoglycan (PGN) and RNA polymerase beta subunit immunoreactivities were observed in all patients, PGN immunodetection was correlated with demyelination and neuroinflammation in MS brains. Principal component analysis revealed that demyelination, PGN and inflammatory gene expression accounted for 86% of the observed variance. Thus, inflammatory demyelination is linked to an organ-specific dysbiosis in MS that could contribute to underlying disease mechanisms.

Resident microorganisms in different tissues of humans and other species are increasingly appreciated as important determinants of health and disease^1^. While the microorganisms comprising human microbiota vary depending on host age, sex and anatomical sites, there is burgeoning recognition that perturbations in organ-specific microbiota are a feature of human diseases^2^. This includes the microbiota colonizing body sites previously considered sterile in the absence of disease including the lower airway and lungs^3^, upper female reproductive tract^4^,^5^, male reproductive tract^6^ and placenta^7^,^8^. Relationships between the gut microbiota and models of MS are being actively pursued^9^,^10^. Blood-derived leukocytes patrol the central nervous system (CNS) in health and this process escalates during systemic inflammation^11^. MS is a common CNS inflammatory demyelinating disorder of unknown etiology that chiefly affects white matter and is driven by activated infiltrating leukocytes^12^. MS exacts a heavy toll on patients’ health, economic status and survival^13^. Microbial associations with MS including viruses and bacteria have been pursued with variable findings^14^,^15^,^16^.

The bacterial cell wall constituent peptidoglycan (PGN) was detected in phagocytes within demyelinating lesions from MS patients and in nonhuman primate models of MS^17^,^18^. PGN might contribute to inflammatory demyelination through engagement of receptor interacting protein kinase (RIPK) 2^19^. Moreover, a component of PGN, muramyl dipeptide, is an established inducer of NOD2 and the NLRP3 inflammasome in human microglia^20^, which is known to influence demyelination^21^. Implantation of endotoxin derived from highly virulent bacteria causes delayed demyelination and innate immune activation^22^. Recent studies demonstrate the presence of bacterium-encoded RNA and DNA sequences, particularly those derived from alpha-Proteobacteria, as well as bacterial proteins, in brains from humans (in the presence or absence of neurological disease), nonhuman primates^23^, rodents^24^ and other species^25^.

Herein, we examined bacterial quantity and genetic diversity in brains from patients with MS and other diseases. Bacterial abundance and molecular diversity were associated with both neuropathology and proinflammatory gene expression in patients with MS, revealing disturbances in human brain microbiota in a disease context.

## Results

### Quantitation and conventional sequencing of brain-derived bacterial 16s and GroEL amplicons

**Figure 1:**
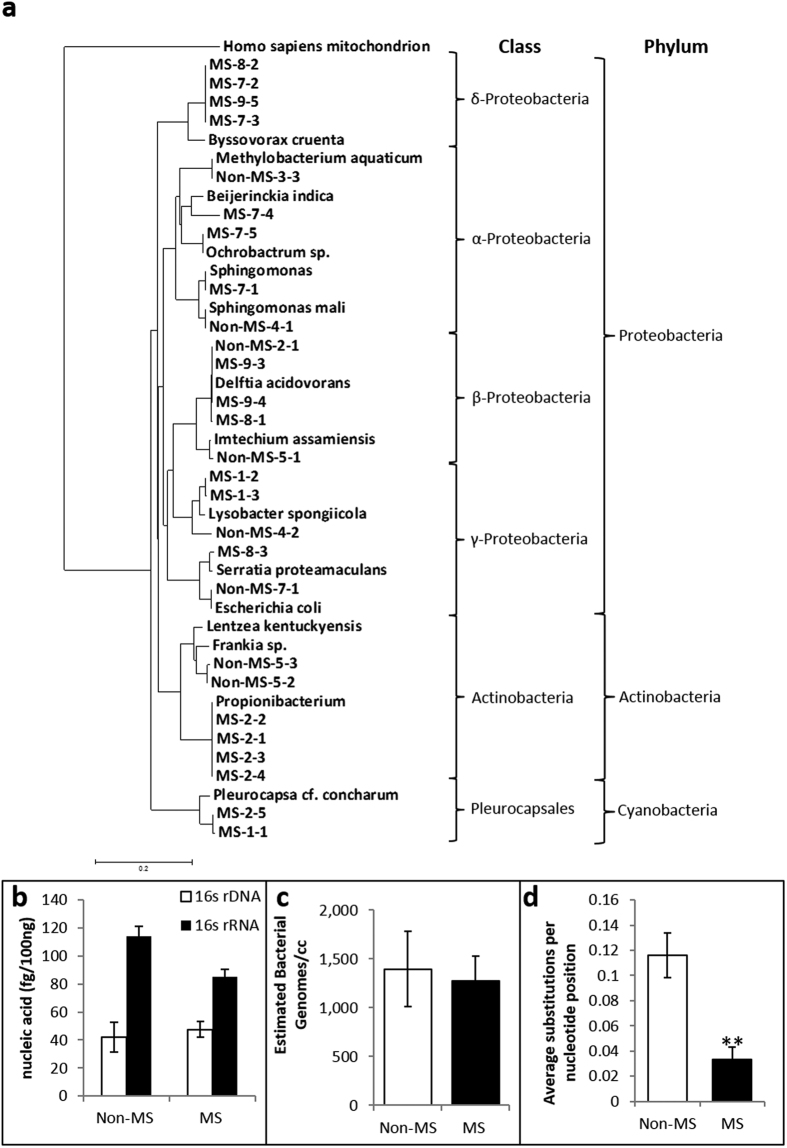
Brain-derived bacterial sequence diversity. (**a**) Phylogenetic analyses of cloned bacterial rRNA V3-V5 sequences showed that cerebral white matter-derived sequences from MS and non-MS patients were complementary to multiple bacterial species depending on individual sequences with a predominance of Proteobacteria-like sequences detected (Clustal W; all bootstrap values ≥ 250/1000) (**b**) Comparison of bacterial 16s rRNA and rDNA sequence levels, amplified from white matter of MS and non-MS brains, revealed increased 16s rRNA levels in all groups relative to rDNA levels. (**c**) Estimates of the bacterial gene (*GroEL*) copy number per cm^3^ of brain tissue. (**d**) Bacterial 16s rRNA molecular diversity, as represented by the mean substitution frequency per nucleotide position within the sequence, was restricted among clones from MS white matter compared to non-MS-derived clones. (Student *t* test, p <0.05).

Bacterial 16s ribosomal rDNA and rRNA V3-V5 sequences (genbank: KX284660- KX284685) were amplified from cerebral white matter of all tested MS (n = 15) and nonMS (n = 15) patients (Table 1) with greater expression of rRNA than rDNA (2–3 fold) in all patients (Fig. 1B). Quantitation of bacterial genomic (*GroEL*) DNA ranged from 1,200–1400 genomes per cm^3^ (tissue) based on single gene copy detection per bacterium in both MS and nonMS white matter (Fig. 1C). Bacterial rRNA sequence analyses of cloned amplicons derived from nonMS white matter displayed greater molecular diversity within multiple clones per sample compared to MS white matter (p < 0.01) (Fig. 1C). Phylogenetic assessment of bacterial sequences encoding the ribosomal 16s rRNA V3–V5 domain cloned from a subset of samples was performed, which revealed alignment of brain-derived bacterial sequences from MS (n = 8) and nonMS (n = 6) patients with diverse bacterial species’ sequences (Fig. 1A). Proteobacteria represented the most abundant phylum in both clinical groups although more cloned bacterial sequences were obtained from MS samples. These findings highlighted the preponderance of bacterial sequences resembling Proteobacterial species in brains with reduced bacterial genomic molecular diversity in MS patients’ white matter.

### RNAseq analyses

**Figure 2:**
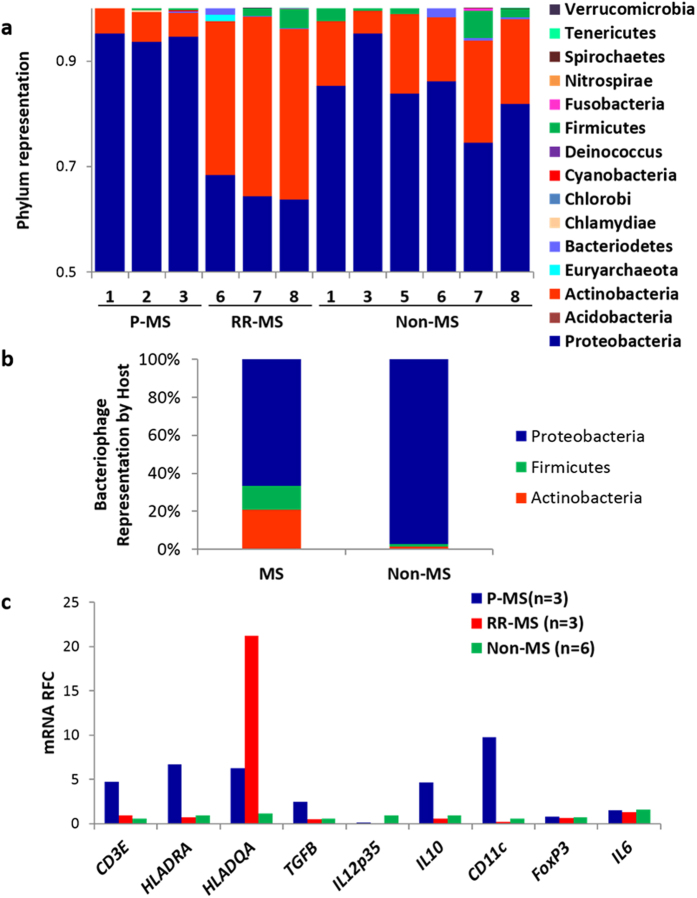
Proteobacteria RNA expression are correlated with host immune responses in MS brains. (**a**) Massively parallel RNA sequencing (RNAseq) of MS and nonMS white matter showed a high proportion of Proteobacteria-like sequences in all human brains. Progressive MS patients (P-MS) displayed limited molecular diversity while relapsing-remitting (RR-MS) patients showed increased Actinobacteria-like sequence detection compared to nonMS patients’ brains. (**b**) Distribution of sequence tags derived from bacteriophage by host phylum shows greater phage diversity in MS white matter. (**c**) Using qRT-PCR host gene expression was measured as relative fold change (RFC) in cerebral white matter from P-MS with low Actinobacteria gene expression (n = 3), RR-MS with high Actinobacteria gene expression (n = 3), compared to nonMS (n = 6); patients with the low Actinobacteria/P-MS group showed increased host immune gene expression.

Massively parallel (deep) sequencing (RNAseq) of total RNA permitted analysis of all RNA sequences in MS (n = 6) and nonMS (n = 6) white matter samples, revealing that bacterial RNA (ribosomal and non-ribosomal) sequences were detected in all nonMS and MS brain specimens, including MS patients with relapsing-remitting disease (receiving disease modifying therapy) (RR-MS, n = 3) and progressive (untreated) MS (P-MS; n = 3) (Fig. 2A). Greater than 60% of bacterial sequences were identified as unambiguously complementary to Proteobacteria sequences. While Actinobacteria was the second most abundant phylum in all cases, there was an enrichment of this phylum in RR-MS (p < 0.001) relative to nonMS white matter with a marked reduction of Actinobacteria sequence proportions in P-MS brains (Fig. 2A). Sequences matching bacteriophages with Proteobacteria hosts predominated in both clinical groups but were overrepresented in nonMS samples (Fig. 2B). The apparent contraction of bacterial molecular diversity within P-MS brains was associated with increased expression of immune genes in P-MS white matter (e.g., *CD3ε, HLA-DRA, IL-10, CD11c*) (Fig. 2C). Proteobacteria sequence abundance was also examined in relation to host gene expression from the same sequencing analyses revealing a positive correlation with multiple groups of genes including *NFKB*-signaling, secondary metabolism, and energy metabolism (Supplementary Fig. 1A). Conversely, expression of genes related to nervous system regulation, gene silencing, and cell proliferation were negatively correlated with Proteobacteria RNA tag abundance (Supplementary Fig. 1B). These observations highlighted the predominance of Protobacteria-encoded RNA sequences in brains from MS and nonMS patients but also displayed variation in bacterial molecular diversity in relation to host immune responses.

### Histopathology, immunohistochemistry and *in situ* hybridization

**Figure 3:**
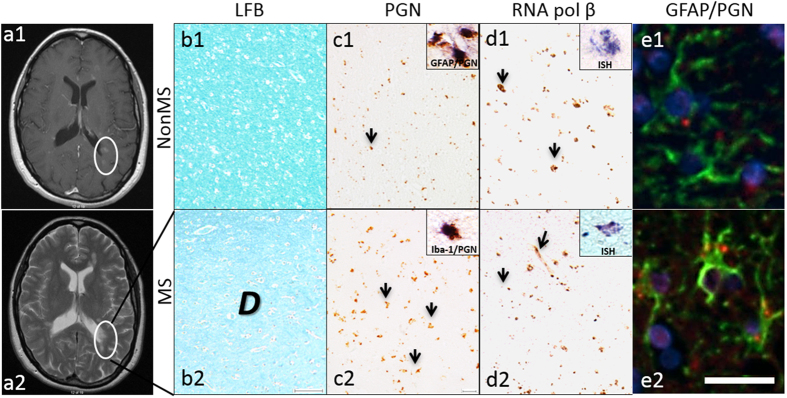
Bacterial immunodetection in MS and nonMS white matter. (**a**) Cranial MRIs from MS patients including gadolinium-enhanced T1 (**a**) and T2 images (**b**) were used to select tissue sections for morphological analyses. NonMS brain showed preserved white matter integrity evidenced by intense LFB staining (b1) while a MS white matter lesion displays reduced LFB detection (b2). (C) In nonMS white matter with PGN (MAB 995) detection was apparent (c1) with similar features in MS normal appearing white matter (c2). (**d**) Bacterial RNA polymerase beta immunoreactivity was present in nonMS (d1) and MS normal appearing white matter (d2); *in situ* hybridization (ISH) detection of bacterial DNA was evident in both nonMS and MS white matter (d1 and d2, insets). (Original magnification: (**a–d**), 10X; e-f, 20X) (Original magnification: 20X) (**e**) Spinning disk confocal images of cells immunolabelled with MAB 995 to PGN (red) GFAP (green) and DAPI (Blue) (Original magnification: 40X; scale bar represents 25 µm).

As MS is a heterogenous disease in terms of clinical features as well as site and type of lesions, we used premortem MRIs to guide the selection of tissue samples when possible (Fig. 3A,B) for neuropathological studies. From brains of MS (n = 12) and nonMS (n = 6) patients, serial brain sections were investigated based on the presence of lesions on MRI that showed gadolinium-enhancement (T1) (Fig. 3A1) and/or evident on T2 images (Fig. 3A2). Strong LFB staining, indicative of intact myelin, was evident in nonMS white matter (Fig. 3B1) but was reduced in demyelinating MS lesions (Fig. 3B2). CD3ε-immunolabeled T cells were occasionally detected in nonMS tissue sections (Supplementary Fig. 2a1) but CD3ε-immunopositive cells were evident in MS lesions (Supplementary Fig. 2a2). CD68-immunopositive brain macrophages were minimally detected in nonMS white matter (Supplementary Fig. 2b1) but were abundant in demyelinating MS lesions (Supplementary Fig. 2b2). Bacterial peptidoglycan (PGN) immunostaining was particulate, detected in sections from nonMS (Fig. 3c1) and MS (Fig. 3c2) brains but appeared to be more concentrated in MS lesions. This was in contrast to the lack of immunostaining apparent with the isotype control (Supplementary Fig. 2c). PGN immunodetection was co-localized with immunoreactivity to the astrocytic protein, glial fibrillary acidic protein (GFAP in both MS and nonMS cases (Fig. 3c1 inset, Fig. 3e1, Supplementary Fig. 3b1,b2) and the microglial protein, Iba-1 (inset Fig. 3c2, Supplementary Fig 3a1,a2, respectively) with approximately 50% of each cell type associated with PGN immunopositive structures (Supplementary Fig. 3c,d). Bacterial RNA polymerase beta subunit immunoreactivity was also evident in both nonMS (Fig. 3d1) and MS (Fig. 3d2) brains together with *in situ* hybridization detection of bacterial 16s rDNA (insets in Fig. 3d1). To verify these findings, neuropathological features were scored by a neuropathologist (JQL), unaware of the slide identity, in the same brain sections from MS and nonMS which showed significantly increased CD3ε and CD68 immunodetection with reduced LFB staining in MS sections compared to nonMS brains. Concurrent semi-quantitative scoring of PGN immunoreactivity within each entire tissue section did not differ between clinical groups (Supplementary Table 2). These findings implied that in nonMS and MS brain tissues, bacterial genomes and proteins were detectable, recapitulating and extending earlier studies of bacterial detection in human brains with and without neurological disease. Despite obvious neuropathological differences, total PGN abundance per brain section was similar in MS and nonMS brains suggesting that overall bacterial burden did not distinguish MS from nonMS patients.

### Quantification of demyelination PGN load and gene expression in tissue sections

**Figure 4:**
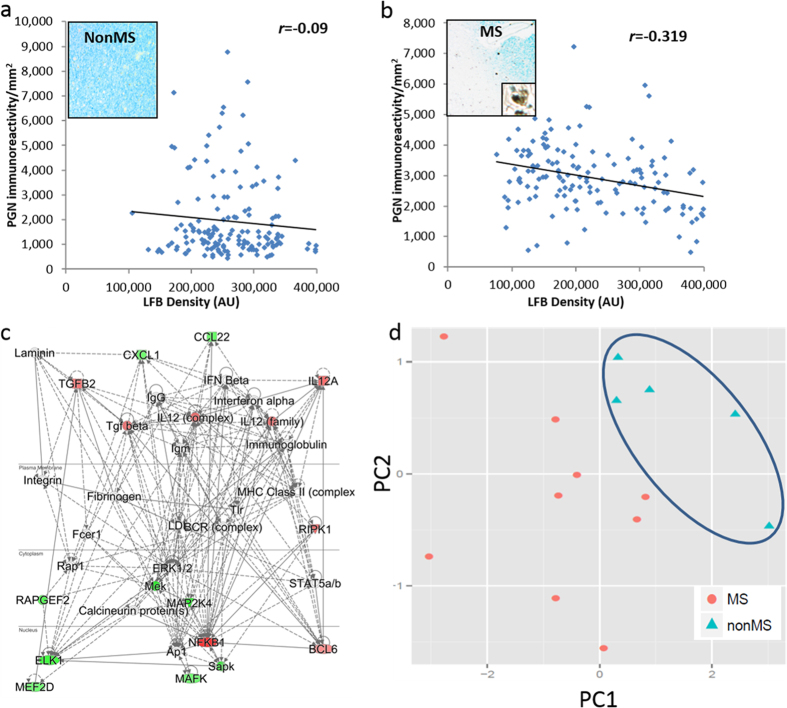
Demyelination is correlated with peptidoglycan detection and immune activation. (**a**) LFB staining (inset) was not correlated with PGN immunodetection in nonMS brain white matter. (**b**) In contrast, reduced LFB (demyelination) was correlated with PGN detection within MS lesions. (**c**) Based on correlations between PGN quantity and Nanostring quantitation of host gene expression on serial white matter sections, a pathway analysis was generated highlighting multiple genes as molecular hubs implicated in MS pathogenesis (IPA = 17). (**d**) Principal components analysis (PCA) including PC1 (Y1 = (0.41)PGN + (0.54) NFKB1 + (0.51)RIPK1 + (0.53)IL12A which accounted for 69% of the variance) and PC2 (Y2 = (0.87)PGN + (0.01)NFKB1 + (0.33)RIPK1 + (0.37)IL12A, which accounted for 17% of the variance); both components accounted for 86% of the original variance and showed clustering of nonMS patients’ white matter in contrast to MS patients’ white matter findings.

The above findings prompted examination of the interactions between demyelination, host innate immune responses and PGN presence. Application of a second anti-PGN monoclonal antibody showed PGN immunolabelling in nonMS (Fig. 4A) and MS (Fig. 4B) brain sections with increased PGN immunoreactivity that was most evident in areas of reduced LFB staining in MS patients. Co-quantitation of myelination and bacterial quantity within each section revealed LFB staining was not correlated with the density of PGN immunolabelling in nonMS brain sections (Fig. 4A) (r = −0.09). In contrast PGN abundance was correlated with decreased LFB staining intensity within MS lesions (Fig. 4B) (r = −0.319). Because of the inverse correlation between LFB intensity and PGN immunolabelling, we examined host gene expression in adjacent serial tissue sections by Nanostring transcript array in sections from each tissue block which disclosed induction of immune genes, *NFKB1, RIPK1* and *IL-12A,* in MS brains and showed the highest correlations with PGN expression. Pathway analyses using these transcript array findings in association with PGN expression revealed an integrated network (Ingenuity Pathway Analysis score = 17) implicating other genes associated with MS pathogenesis (e.g., MHC Class II, ERK1/2, and immunoglobulin) as molecular hubs (Fig. 4C). Principal component analysis (PCA) including linked PGN and transcript (*NFKB1, RIPK1* and *IL-12A*) expression as first (69% of variance) and second components (17% of variance) revealed clustering of nonMS white matter samples (Fig. 4D) while MS samples were scattered, reflecting the heterogeneity of disease severity. These observations indicated that a key bacterial component (PGN) was concentrated within demyelinating MS lesions in association with innate immune gene activation.

## Discussion

The current study shows the presence of bacterial RNA and DNA sequences and proteins in human brain which are disrupted in conjunction with inflammatory demyelination in patients with MS. Proteobacteria represented the chief bacterial phylum detected in human brain with restricted molecular diversity in MS brains despite the increased density of bacterial glycoproteins within demyelinating lesions. Evidence for bacterial presence in human brains was verified by using several monoclonal antibodies that recognized specific bacterial proteins, *in situ* hybridization, PCR amplification and cloning of bacterial RNA and DNA sequences together with massively parallel sequencing of brain-derived RNA. The presence and type of bacteria in brain was associated with host immune gene expression, which were apparent using different methods. These findings indicated a strong interaction between bacterial presence and host responses involving NFκB-related signaling in demyelinating lesions, which is a pivotal pathway in neuroinflammation and MS pathogenesis^26^.

As contamination of tissue specimens and experimental tools was a paramount concern in the present study, extensive precautions were implemented to mitigate this concern, given the relative low levels of bacterial burden observed in the present studies. Phylogenetic analyses of PCR-derived sequences displayed similarities to Propionibacterium, a common cutaneous and nasopharyngeal bacterium in a single MS patient which might reflect contamination during the autopsy process or alternatively *in vivo* transport from the nasopharynx via the cribriform plate, as reported for other bacteria^27^. However, the correlations between peptidoglycan abundance and host neuroimmune responses, using both Nanostring and deep sequencing, argues against contamination, particularly the consistent preponderance of NFκB-associated transcriptional observations (Figs 2 and 4, Supplementary Fig. 1A). Moreover, the presence of viruses in the brain inducing local immune responses was unlikely here because of the relative paucity of detectable viral genomes (Supplementary Table 3). Reduced molecular diversity among MS brain-derived 16s V3-V5 rRNA sequences as well as the increased density of PGN immunoreactivity associated with demyelination are also at odds with contamination as the sole explanation for the present observations. The present studies revealed the ratio of bacterium-encoded 16s rDNA to rRNA in matched brain samples to be ~1:2 in both white matter (and cortex, data not shown) with bacterial numbers of 1200–1400 genomes/cm^3^ suggesting both bacterial burden and replication were low compared to active pathogenic infections in other tissues. The low bacterial rDNA:rRNA ratio and burden are not surprising given the sensitivity of the brain to bacterial molecules (e.g., endotoxin), which at high levels can lead to adverse effects on brain tissue. The reduced molecular diversity of bacterial RNA sequences in MS brains implies an overgrowth in select bacteria, perhaps Proteobacteria, as indicated by the current sequencing data (Fig. 2) from progressive MS patients, which was diversified in relapsing-remitting MS patients receiving disease modifying therapies. These results recapitulate changes in microbial populations in other diseased organs and are reminiscent of a dysbiosis that can be resolved by increasing microbial diversity. The differential distribution of individual bacterial phyla consistently associated with clinical phenotype and immune activation, observed across multiple lots of reagents, collectively support the specificity of the current findings.

The detection of bacterial DNA and RNA sequences, proteins and cell wall components in human brains raises the question of how bacteria might enter the brain. The bacterial sequences detected in the present studies of human brain resemble those of environmental (soil-derived) bacteria^28^, largely without human disease associations. Phagocytes including macrophages, neutrophils and dendritic cells can engulf live microbes or microbial compounds at different mucosal sites^29^. Polymicrobial species have been detected in human blood, particularly in leucocytes including neutrophils and macrophages^30^. In rats with experimental autoimmune encephalomyelitis, leukocytes enter the brain following activation in the lung^31^, suggesting that lung epithelium also provides an interface for microbial translocation. Given that the present bacterial species’ sequences are similar to environmental bacteria, inhalation and phagocytosis with ensuing trafficking to the brain is a plausible route of CNS entry. The presence of bacteria or their components (e.g., PGN), even in a quiescent state and in low copy numbers in the brain, could exert effects on neurocognitive functions, inflammatory gene expression, and perhaps on neural cell (e.g., oligodendrocyte) survival. Potential consequences of resident bacteria in brain include altered myelin viability and repair as well as activation of host inflammatory genes with pathogenic or protective effects^32^. These effects could be consequences of colonization by viable bacteria or the presence of bacterial PAMPs from non-viable bacteria. Our previous study^23^ in which we successfully transmitted human brain-derived bacteria to immunocompromised mice suggests that some of these organisms are viable as the persistence of residual RNA is not sufficient to explain the finding given the duration of those studies. Identifying the individual bacterial species and their impact on inflammatory demyelination might yield insights into MS prevention and treatment. Indeed, recent studies of minocycline treatment of MS (clinicaltrials.gov number NCT00666887) could also be interpreted within the context of the current study. Similarly the putative neurotoxic effects of antibiotics might be related to their actions on microbiota located in the brain (or gut) with potential adverse consequences for gut-brain interactions^33^,^34^. Further delineation of the individual bacterial species in brain and other organs that contribute to neurological disease (or health) are warranted because they might offer new therapeutic approaches or targets for inflammatory degenerative neurological diseases.

## Methods

### Ethics Statement

The use of autopsied brain tissues with associated clinical data (age, sex, MS phenotype, EDSS, duration of disease) was approved by the University of Alberta Human Research Ethics Board (Biomedical, Protocol number 2291). Written informed consents were signed before or at the collection time. The protocols for obtaining post-mortem brain samples were performed in accordance with the Canadian Association of Pathologists policy statement and guidelines for the ethical use of human tissue in research and all federal and institutional guidelines with special respect for the confidentiality of the donor’s identity. All frozen tissues were stored at −80 °C at the time of autopsy. Brain tissues were obtained from MS patients including relapsing-remitting (RR-MS) and progressive (primary and secondary) (P-MS) or disease controls (nonMS) (Table 1)^35^,^36^,^37^.

### Specificity and control measures

In view of the study’s overall aim, explicit attention was paid to issues of contamination and specificity. Brain tissues from MS and nonMS patients was obtained by aseptic collection at autopsy into sterile vessels with immediate flash freezing. Patient brains were collected at separate sites in Canada (Winnipeg, Calgary, Edmonton and Vancouver) reducing the probability of shared contaminating microorganisms. All post-collection tissue manipulation was performed in decontaminated biosafety hoods with autoclaved or chemically decontaminated tools and no sampling was performed from the exposed surface of tissues. All assays including RNA and DNA extractions contained water controls that were carried forward through all subsequent steps and each new step added an additional water control including cloning and Sanger sequencing. All data in which any steps showed evidence of reagent contamination were excluded and the previous steps were performed with new lots of reagents. All amplification steps were set up in areas and with equipment treated with DNase and RNase inhibitor (Molecular BioProducts, San Diego CA, USA). All post-amplification steps were performed in a discrete space with separate equipment.

### Immunohistochemistry, histochemistry and quantitation

Formalin-fixed paraffin-embedded brain was processed and brain sections (Two regions from MS case 9, 11, 12, 16–18 and two from nonMS cases 16–21) (10 μm) were stained with Luxol fast blue (LFB) to visualize myelin. In addition, serial brain sections were immuno-labelled with antibodies to bacterial and host proteins. Immunocytochemistry was performed with two mouse anti-PGN antibodies (MAB995, generated against *Streptococcus mutans* peptidoglycan, Chemicon, Temecula, CA; MAB 2E9, mouse IgG3, generated against a human gut PGN preparation at Erasmus MC Rotterdam, Netherlands^18^), and mouse anti-RNA polymerase beta subunit, mouse IgG1, generated against *E*. *coli* recombinant protein (Neoclone, Madison, WI), rabbit anti-glial fibrillary acidic protein (GFAP) (Dako, Carpenteria CA) and rabbit anti-Iba-1 (Wako Pure Chemical Industries Ltd., Osaka Japan), Microglia/macrophages were detected with a rabbit polyclonal anti-CD68 and T-cells by anti-CD3ε which was quantified as previously reported^38^ together with appropriate secondary antibodies for single or double immunolabeling^35^,^36^,^37^.

### Immunofluorescence

Slides were deparaffinized by incubation for 1 hour at 60 degrees followed by one 10 minute and 2 five minute incubations in xylene baths through decreasing concentrations of ethanol to distilled water. Antigen retrieval was performed by boiling in 10 mM sodium citrate (pH 6.0) 1hr. Slides were blocked with HHFH buffer (1 mM HEPES buffer, 2% (v/v) horse serum, 5% (v/v) FBS, 0.1% (w/v) sodium azide in Hank’s balanced salt solution (HBSS)) for 4 hours at room temperature. Slides were incubated with a cocktail of rabbit anti-GFAP or anti-Iba-1 (1:400), MAB995 against peptidoglycan (1:150) overnight at four degrees Primary antibody was removed by three 5 min PBS washes and slides were incubated for three minutes in 0.22 micron filtered 1% (w/v) Sudan black in 70% ethanol and washed an additional 3 times in PBS. A cocktail of 1:500 Alexa 488 goat anti rabbit IgG, Alexa 568 goat anti mouse IgG for two hours, washed three times in PBS stained with DAPI for 10 minutes, washed 3 times in PBS and mounted with Prolong gold antifade reagent. Slides were imaged with wave fx spinning disc confocal microscope (Zeiss). Total GFAP and Iba-1 positive cells were counted in ten fields per case at 20X and the number of cells with attached peptidoglycan immune positive particles and internalized particles were counted.

### Semi-quantitative scoring of neuropathology

Neuropathological scoring was performed as modified from past studies^38^,^39^. Scoring of LFB (0.1%) staining, CD68 (Dako, Carpenteria, CA), CD3 (Dako, Carpenteria, CA) and PGN (MAB 995) immunoreactivity in serial brain sections was performed as follows (modified from^38^,^40^) with differences in staining or immunoreactivity defined relative to normal appearing white matter in terms of area and/or intensity (10X magnification). Decreased density of LFB staining for myelin was scored in (original magnification 10 × 10) as follows: 0, normal to minimal decrease; 1, identifiable to 50% of decrease; 2, more than 50% of decrease to little preservation; 3, complete loss. Decrease in LFB staining was defined by reduction of tissue reactivity for LFB, compared to normal appearing white matter (NAWM), in terms of its size and/or intensity. The scores of every 10 consecutive fields were summed, and then 5–10 (depending on the tissue section sizes) sets were averaged for analysis. CD68+ and CD3ε+ cells were assessed by scoring the frequency of positive cells in a 10 × 10 (original magnification) field: 0, none; 1, sparse; 2, scattered; 3, frequent. Positive cells were identified as those with visible immunoreactivity within the cytoplasm. The scores of every 10 consecutive fields were summed, and then 3 summed sets were averaged for analysis.

PGN immunostaining was scored based on the following criteria: 0, none; 1, weak and focal; 2, strong but focal (50% or less of the entire tissue section); 3, strong and diffuse (more than 50% of the entire tissue section), overall sparse to scattered; 4 strong and diffuse (more than 50% of the entire tissue section), overall frequent.

To quantify LFB and peptidoglycan immunodetection simultaneously images from LFB/anti-peptidoglycan double-labelled slides were divided into three fields. LFB staining was quantified using Image J Fiji^41^ and PGN immunolabelled particles (brown) in 4 pixels or greater in size (magnification X20) and separated by two or more pixels of another color were counted as immunopositive signals in coincident fields.

### *In situ* hybridization

ISH performed as previous^23^. In brief, slides were de-paraffinized, rehydrated then treated with 10 mg/mL hen egg lysozyme (Sigma, Oakville, ON, Canada) for 20 minutes followed by treatment with 100 μg/mL proteinase K in buffer for 10 minutes at 37 °C then washing and dehydration. 150 μL of pre-warmed 2 ng/μL double DIG labeled EUB338 (*GCTGCCTCCCGTAGGAGT*) probe targeting bacterial 16s rDNA or scrambled probe in hybridization buffer (25 mM Tris-HCl, 100 mM NaCl, 0.5% SDS pH 9) was applied to each sample and incubated at 50 °C for 90 minutes (Sigma, Oakville, ON, Canada)^42^. Slides were then washed in six rapid changes of 50 °C wash buffer (10 mM Tris, pH 9.0, 1 mM EDTA). The samples were then blocked first for 30 min. with levamisole, then for 1 hr with Odyssey blocking buffer (LiCor, Lincoln, NE, USA). A 1∶200 dilution of AP (Alkaline Phosphatase) conjugated sheep anti-DIG Fab’ fragments (Roche, Mannheim, Germany) was applied to the slides and incubated o/n at 4 °C. The slides were washed 3 times in PBS and incubated for 2 hours in the dark at 30 °C with AP substrate (Roche, Mannheim, Germany), then washed 3 times in PBS, mounted and imaged.

### DNA and RNA preparation

Total RNA was extracted using the Qiagen RNeasy and DNA was extracted using the Qiagen DNeasy blood and tissue kit from frozen normal appearing white matter in parallel with Ultrapure Rnase- and DNase-free water (Life technologies, 10977-015) to monitor for reagent contamination according to the manufacturer’s protocol (Qiagen, Toronto, ON) (see Supplemental Material and Methods). A subset of RNA (MS 1–3, 6–8 nonMS 1, 3, 5–8) selected based on maximum RNA quality, was subjected to massively parallel sequencing as described previously^23^.

### cDNA synthesis and bacterial RNA and DNA quantitation

First strand cDNA synthesis and RT-PCR was performed as previously described^43^. Semi-quantitative RT-PCR was performed for several host response genes and viruses using primers described in Supplementary Table 1. The quantity of bacterially-encoded 16s rRNA, rDNA and GroEl was determined using the primers described in Supplementary Table 1 on samples from MS cases 1–15 and nonMS cases 1–15 by comparison to standard curves generated from pGem-T clones of the *E. coli* 16s rDNA and *GroEL*, respectively. The number of bacterial genomes was estimated based on the quantity of *GroEL-*encoding DNA sequences^44^) detected per mass (g) of tissue from which the gDNA was extracted, assuming a single *GroEL* copy per genome.

16s rRNA clones were generated from MS cases 1–8 and NonMS cases 2–7 using the pGEM T-easy vector system (Promega, Madison, WI) and sequenced in both directions with T7 and M13R primers using Big Dye terminator cycle sequencing kit (Thermo Fisher, Foster City, CA) and analyzed^23^.

### Nanostring analyses

RNA was extracted from serial 10 μm brain sections (per case) and analyzed with the nCounter human inflammation panel containing 190 genes as per the manufacturer’s instructions (Nanostring Technologies, Seattle, WA).

### Bioinformatics

Gene ontology analysis was performed using DAVID Bioinformatic resources 6.7 Functional Annotation^45^,^46^ (http://david.abcc.ncifcrf.gov/). Gene network analysis of transcripts identified by Nanostring shown to be correlated with PGN immunostaining was performed using the network tool in Ingenuity pathway analysis (Ingenuity systems, www.ingenuity.com). Alignment of RNAseq-derived bacterial sequences was performed (Novoalign)^23^.

### Statistics

Univariate and correlation analyses were performed using SPSS using the Student *t and Tukey-Kramer* tests (2-tailed) and Spearman correlation. Principal components analysis (PCA) was performed to investigate multivariate correlations within the data including PGN immunoreactivity, Nanostring, LFB staining within individual patient groups (R Core Team, 2015, http://www.R-project.org).

## Additional Information

**Accession Codes**: Genbank accession numbers KX284660- KX284685

**How to cite this article**: Branton, W. G. *et al*. Brain microbiota disruption within inflammatory demyelinating lesions in multiple sclerosis. *Sci. Rep.* **6**, 37344; doi: 10.1038/srep37344 (2016).

**Publisher's note:** Springer Nature remains neutral with regard to jurisdictional claims in published maps and institutional affiliations.

## Supplementary Material

Supplementary Information

## Figures and Tables

**Table 1:**
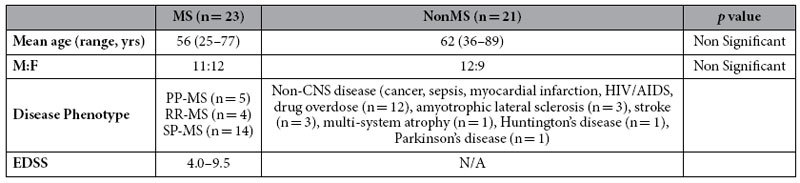
Clinical features of MS and nonMS patients.

## References

[b1] Lozupone C. . Widespread colonization of the lung by Tropheryma whipplei in HIV infection. Am J Respir Crit Care Med 187, 1110–1117 (2013). 23392441 10.1164/rccm.201211-2145OC PMC3734615

[b2] Hugon P. . A comprehensive repertoire of prokaryotic species identified in human beings. Lancet Infect Dis. 15(10), 1211–1219, doi: 10.1016/S1473-3099(15)00293-Oct5 (2015). 26311042

[b3] Dickson R. P. . Spatial Variation in the Healthy Human Lung Microbiome and the Adapted Island Model of Lung Biogeography. Ann Am Thorac Soc 12, 821–830 (2015). 25803243 10.1513/AnnalsATS.201501-029OC PMC4590020

[b4] Verstraelen H. . Characterisation of the human uterine microbiome in non-pregnant women through deep sequencing of the V1–2 region of the 16S rRNA gene. PeerJ 4, e1602 (2016). 26823997 10.7717/peerj.1602 PMC4730988

[b5] Mitchell C. M. . Colonization of the upper genital tract by vaginal bacterial species in nonpregnant women. Am J Obstet Gynecol 212, 611 e611–e619 (2015). 10.1016/j.ajog.2014.11.043 PMC4754962 25524398

[b6] Javurek A. B. . Discovery of a Novel Seminal Fluid Microbiome and Influence of Estrogen Receptor Alpha Genetic Status. Sci Rep 6, 23027 (2016). 26971397 10.1038/srep23027 PMC4789797

[b7] Aagaard K. . The placenta harbors a unique microbiome. Sci Transl Med 6, 237ra265 (2014). 10.1126/scitranslmed.3008599 PMC4929217 24848255

[b8] Onderdonk A. B. . Colonization of second-trimester placenta parenchyma. Am J Obstet Gynecol 199, 52 e51–52 e10 (2008). 18313635 10.1016/j.ajog.2007.11.068 PMC2827873

[b9] Wang Y. . An intestinal commensal symbiosis factor controls neuroinflammation via TLR2-mediated CD39 signalling. Nat Commun 5, 4432 (2014). 25043484 10.1038/ncomms5432 PMC4118494

[b10] Berer K. . Commensal microbiota and myelin autoantigen cooperate to trigger autoimmune demyelination. Nature 479, 538–541 (2011). 22031325 10.1038/nature10554

[b11] Ousman S. S. & Kubes P. Immune surveillance in the central nervous system. Nat Neurosci 15, 1096–1101 (2012). 22837040 10.1038/nn.3161 PMC7097282

[b12] Ciccarelli O. . Pathogenesis of multiple sclerosis: insights from molecular and metabolic imaging. Lancet Neurol 13, 807–822 (2014). 25008549 10.1016/S1474-4422(14)70101-2

[b13] Koch-Henriksen N. & Sorensen P. S. The changing demographic pattern of multiple sclerosis epidemiology. Lancet Neurol 9, 520–532 (2010). 20398859 10.1016/S1474-4422(10)70064-8

[b14] M Mayne J. J. Latent and activated brain flore: human herpes virus, endogenous retroviruses,coronaviruses and Chlamydia and their role in neurological disease. In Emerging Neurological Infections (ed. Johnson C.P.a.R.T.) 363–396 (Taylor and Francis, 2005).

[b15] Sriram S. . Chlamydia pneumoniae infection of the central nervous system in multiple sclerosis [see comments]. Ann Neurol 46, 6–14 (1999). 10401775

[b16] Soldan S. S. . Association of human herpes virus 6 (HHV-6) with multiple sclerosis: increased IgM response to HHV-6 early antigen and detection of serum HHV-6 DNA [see comments]. Nature medicine 3, 1394–1397 (1997). 10.1038/nm1297-1394 9396611

[b17] Schrijver I. A. . Bacterial peptidoglycan and immune reactivity in the central nervous system in multiple sclerosis. Brain 124, 1544–1554 (2001). 11459746 10.1093/brain/124.8.1544

[b18] Visser L. . Phagocytes containing a disease-promoting Toll-like receptor/Nod ligand are present in the brain during demyelinating disease in primates. Am J Pathol 169, 1671–1685 (2006). 17071591 10.2353/ajpath.2006.060143 PMC1780210

[b19] Shaw P. J. . Signaling via the RIP2 adaptor protein in central nervous system-infiltrating dendritic cells promotes inflammation and autoimmunity. Immunity 34, 75–84. 21236705 10.1016/j.immuni.2010.12.015 PMC3057380

[b20] Ramaswamy V. . Inflammasome induction in Rasmussen’s encephalitis: cortical and associated white matter pathogenesis. J Neuroinflammation 10, 152 (2014). 10.1186/1742-2094-10-152 PMC3881507 24330827

[b21] Guo H., Callaway J. B. & Ting J. P. Inflammasomes: mechanism of action, role in disease, and therapeutics. Nature medicine 21, 677–687 (2015). 10.1038/nm.3893 PMC4519035 26121197

[b22] Felts P. A. . Inflammation and primary demyelination induced by the intraspinal injection of lipopolysaccharide. Brain 128, 1649–1666 (2005). 15872019 10.1093/brain/awh516 PMC7109778

[b23] Branton W. G. . Brain Microbial Populations in HIV/AIDS: alpha-Proteobacteria Predominate Independent of Host Immune Status. PLoS One 8, e54673 (2013). 23355888 10.1371/journal.pone.0054673 PMC3552853

[b24] Lluch J. . The Characterization of Novel Tissue Microbiota Using an Optimized 16S Metagenomic Sequencing Pipeline. PLoS One 10, e0142334 (2015). 26544955 10.1371/journal.pone.0142334 PMC4636327

[b25] Rosales S. M. & Thurber R. V. Brain Meta-Transcriptomics from Harbor Seals to Infer the Role of the Microbiome and Virome in a Stranding Event. PLoS One 10, e0143944 (2015). 26630132 10.1371/journal.pone.0143944 PMC4668051

[b26] Frohman E. M., Racke M. K. & Raine C. S. Multiple sclerosis–the plaque and its pathogenesis. N Engl J Med 354, 942–955 (2006). 16510748 10.1056/NEJMra052130

[b27] van Ginkel F. W. . Pneumococcal carriage results in ganglioside-mediated olfactory tissue infection. Proc Natl Acad Sci USA 100, 14363–14367 (2003). 14610280 10.1073/pnas.2235844100 PMC283597

[b28] Hultman J. . Multi-omics of permafrost, active layer and thermokarst bog soil microbiomes. Nature 521, 208–212 (2015). 25739499 10.1038/nature14238

[b29] Powell J. J. . An endogenous nanomineral chaperones luminal antigen and peptidoglycan to intestinal immune cells. Nat Nanotechnol 10, 361–369. 25751305 10.1038/nnano.2015.19 PMC4404757

[b30] Gyarmati P. . Bacterial Landscape of Bloodstream Infections in Neutropenic Patients via High Throughput Sequencing. PLoS One 10, e0135756 (2015). 26270467 10.1371/journal.pone.0135756 PMC4536222

[b31] Odoardi F. . T cells become licensed in the lung to enter the central nervous system. Nature 488, 675–679. 22914092 10.1038/nature11337

[b32] Yong V. W. & Rivest S. Taking advantage of the systemic immune system to cure brain diseases. Neuron 64, 55–60 (2009). 19840549 10.1016/j.neuron.2009.09.035

[b33] Collins S. M., Surette M. & Bercik P. The interplay between the intestinal microbiota and the brain. Nat Rev Microbiol 10, 735–742 (2012). 23000955 10.1038/nrmicro2876

[b34] Bhattacharyya S., Darby R. R., Raibagkar P., Gonzalez Castro L. N. & Berkowitz A. L. Antibiotic-associated encephalopathy. Neurology 86, 963–971 (2016). 26888997 10.1212/WNL.0000000000002455

[b35] Tsutsui S. . A1 Adenosine Receptor Upregulation and Activation Attenuates Neuroinflammation and Demyelination in a Model of Multiple Sclerosis. J Neurosci. 24, 1521–1529. (2004). 14960625 10.1523/JNEUROSCI.4271-03.2004 PMC6730323

[b36] Noorbakhsh F. . Proteinase-activated receptor 2 modulates neuroinflammation in experimental autoimmune encephalomyelitis and multiple sclerosis. J Exp Med 203, 425–435 (2006). 16476770 10.1084/jem.20052148 PMC2118197

[b37] Deslauriers A. M. . Neuroinflammation and endoplasmic reticulum stress are coregulated by crocin to prevent demyelination and neurodegeneration. J Immunol 187, 4788–4799 (2011). 21964030 10.4049/jimmunol.1004111

[b38] Lu J. Q. . Neuroinflammation and demyelination in multiple sclerosis after allogeneic hematopoietic stem cell transplantation. Arch Neurol 67, 716–722. 20558390 10.1001/archneurol.2010.117

[b39] Lu J. Q., Power C., Blevins G., Giuliani F. & Yong V. W. The regulation of reactive changes around multiple sclerosis lesions by phosphorylated signal transducer and activator of transcription. Journal of neuropathology and experimental neurology 72, 1135–1144 (2013). 24226263 10.1097/NEN.0000000000000011

[b40] Assessment, diagnosis, and treatment of HIV-associated neurocognitive disorder: a consensus report of the mind exchange program. Clin Infect Dis 56, 1004–1017 (2013). 23175555 10.1093/cid/cis975 PMC3657494

[b41] Schindelin J. . Fiji: an open-source platform for biological-image analysis. Nat Methods 9, 676–682. 22743772 10.1038/nmeth.2019 PMC3855844

[b42] AMANN R. I. *In situ* identification of micro-organisms by whole cell hybridization with rRNA-targeted nucleic acid probes. *In:* Molecular Microbial Ecology Manual (Akkeman A. D. C., Van Elsas J. D., De Bruigin F. J., eds.) (1995).

[b43] Power C. . Intracerebral hemorrhage induces macrophage activation and matrix metalloproteinases. Ann Neurol 53, 731–742 (2003). 12783419 10.1002/ana.10553

[b44] Hill J. E., Town J. R. & Hemmingsen S. M. Improved template representation in cpn60 polymerase chain reaction (PCR) product libraries generated from complex templates by application of a specific mixture of PCR primers. Environmental microbiology 8, 741–746 (2006). 16584485 10.1111/j.1462-2920.2005.00944.x

[b45] Huang da W., Sherman B. T. & Lempicki R. A. Systematic and integrative analysis of large gene lists using DAVID bioinformatics resources. Nat Protoc 4, 44–57 (2009). 19131956 10.1038/nprot.2008.211

[b46] Huang da W., Sherman B. T. & Lempicki R. A. Bioinformatics enrichment tools: paths toward the comprehensive functional analysis of large gene lists. Nucleic acids research 37, 1–13 (2009). 19033363 10.1093/nar/gkn923 PMC2615629

