# Supplementary material for: Brain microbiota disruption within inflammatory demyelinating lesions in multiple sclerosis
Source: Sci Rep. 2016 Nov 28;6:37344. doi: 10.1038/srep37344 (PMC5125007; doi:10.1038/srep37344)
Supplement: Supplementary Information [file srep37344-s1.pdf]

Brain microbiota disruption within inflammatory demyelinating lesions in multiple sclerosis

W.G. Branton MSc[1,7], J.Q. Lu MD PhD[2,7] , M.G. Surette PhD [4], R.A. Holt PhD [5], J. Lind PhD[3], J.D. Laman PhD[6] and C. Power MD[1,3,7]

Departments of Medicine[1], Laboratory Medicine & Pathology[2], and Psychiatry[3], Multiple Sclerosis Centre[7], University of Alberta, Edmonton AB; Department of Medicine[4], McMaster University, Hamilton ON; Genome Sciences Centre[5], Vancouver BC; Department of Neurosciences, Section of Medical Physiology, Faculty of Medical Sciences[6], University Medical Center Groningen, University of Groningen, Groningen NL.

Correspondence to C. Power MD, Department of Medicine (Neurology), 6-11 Heritage Medical Research Centre, University of Alberta, Edmonton, AB, Canada, T6G2S2. Email: cp9@ualberta.ca, Tel: +017804071938, Fax: +017804071984

Supplementary Figure 1

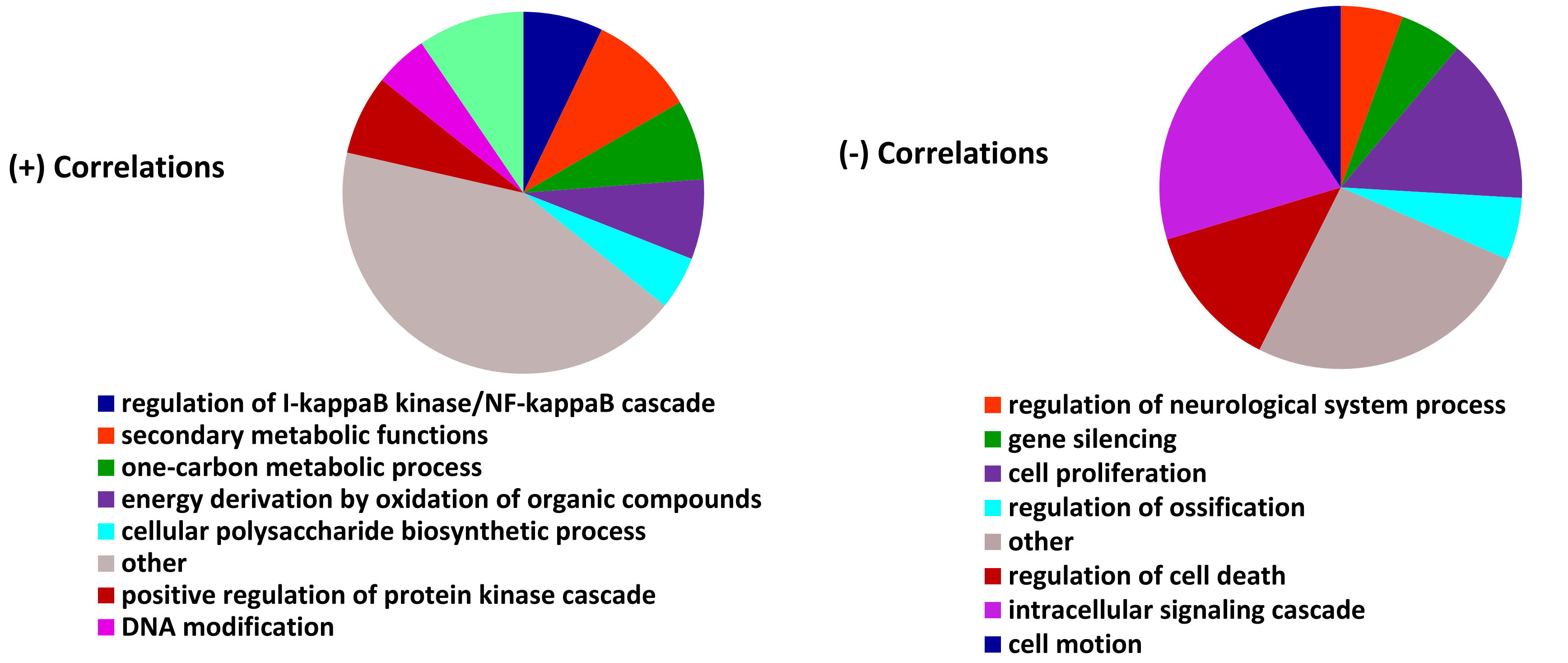

Supplementary Figure 1: ***Host gene groups associated with bacterial gene expression*****.** (A) The quantity of transcripts belonging to multiple human gene groups were positively correlated with the abundance of alpha-Proteobacteria sequences including NFκB-associated genes. (B) In contrast, several groups of genes implicated in cell gene and growth silencing were negatively correlated with alpha-Proteobacteria sequence levels.

Supplementary Figure 2

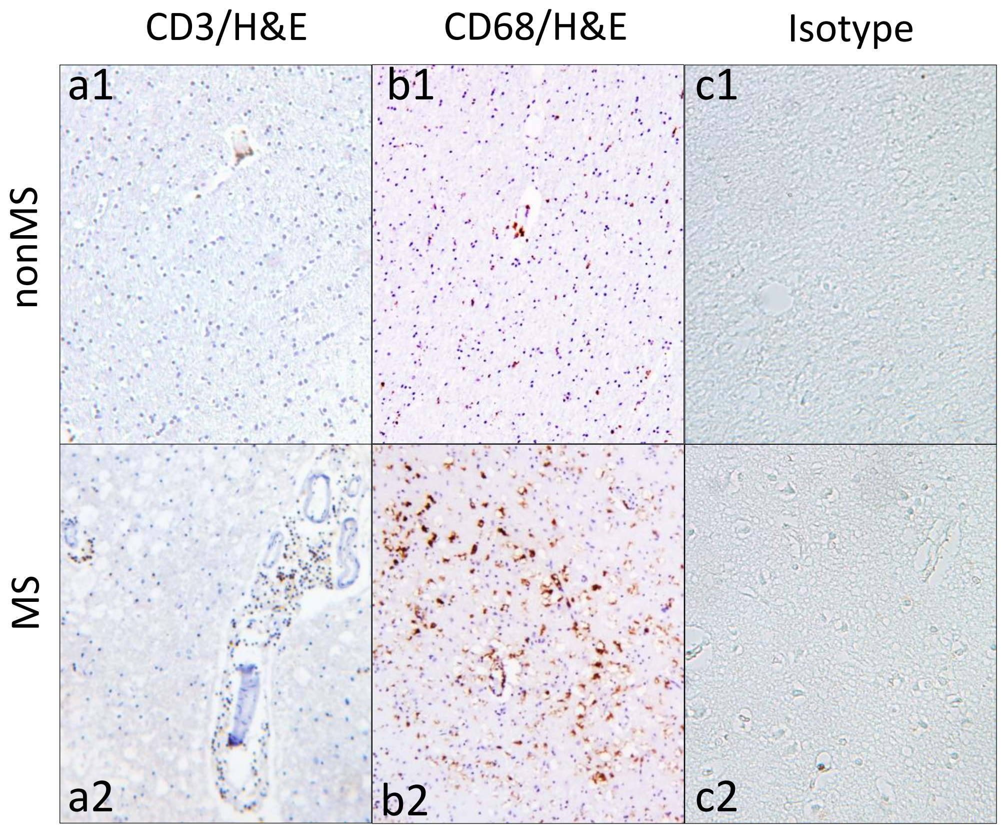

Supplementary Figure 2: ***Immunohistochemical characterization of MS lesion.*** (A) CD3 immunostaining (DAB: brown). (B) CD68 immunostaining (DAB: brown). (C) IgG1 isotype control for monoclonal antibodies used in this study.

Supplementary Figure 3

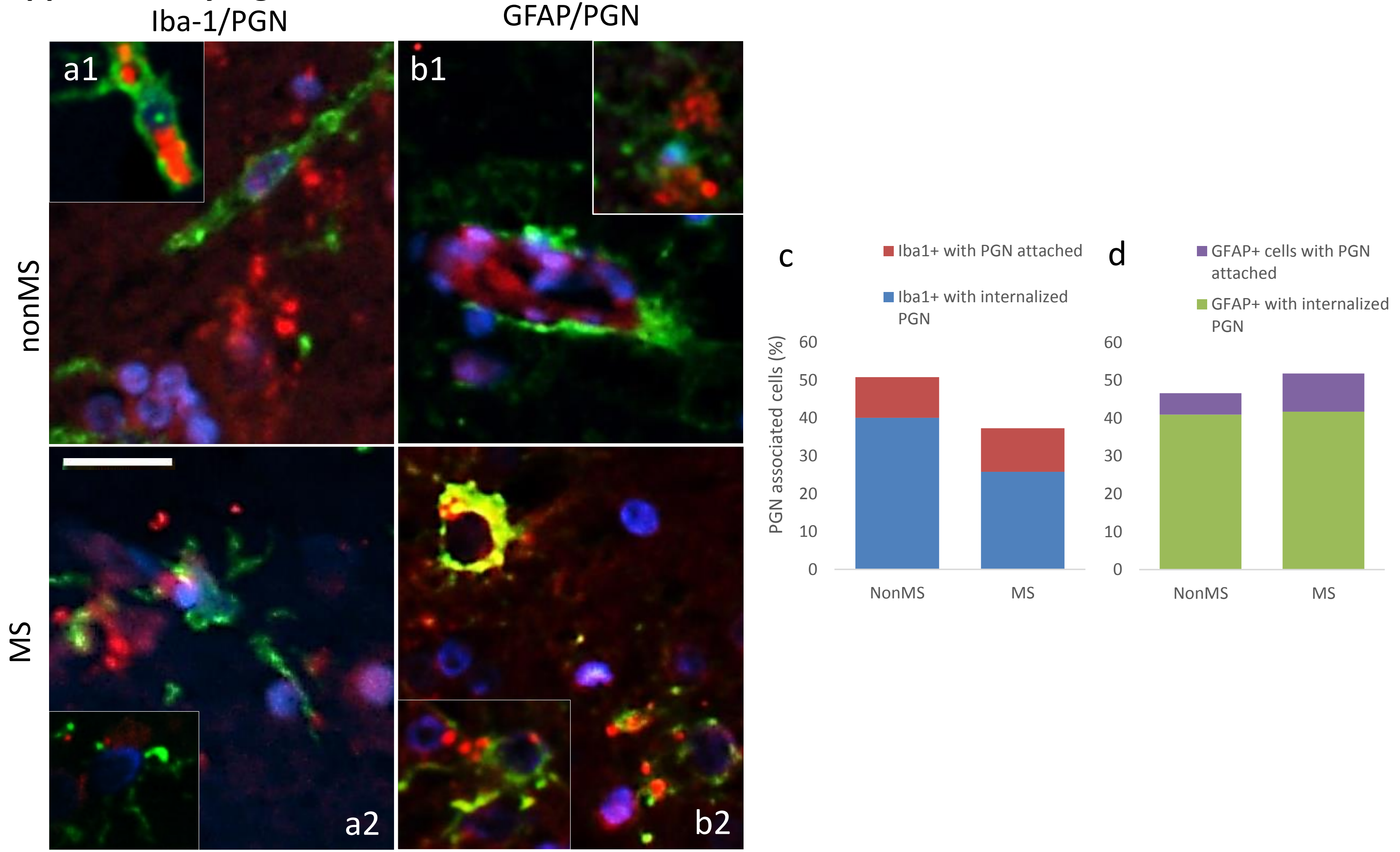

Supplementary Figure 3: Immunofluorescent labelling of PGN and glia. a) Tissue sections from nonMS (a1) and MS (a2) patients immunolabeled with rabbit ani-Iba1 (green), mouse anti-PGN (Mab 995) (red) and DAPI (Blue) b) Tissue sections from nonMS (b1) and MS (b2) patients labeled with rabbit ani-GFAP (green), mouse anti-PGN (Mab 995) (red) and DAPI (Blue) c) Mean percentage of Iba1^{+} with attached or internalized peptidoglycan (PGN). d) Mean percentage of GFAP^{+} cells with attached or internalized peptidoglycan (PGN) Values represent the mean per case percentage from 10 non-adjacent fields of view (n=4).

Supplementary Table 1: Primers used for semi-quantitative RT-PCR analysis and PCR detection of viruses

Primer	Sequence	Primer	Sequence
Hu HLA-DRA fwd	GGACAAAGCCAACCTGGAAA	HSV-1 fwd 1	TTCTCGTTCCTCACTGCCTCCC
Hu HLA-DRA rev	AGGACGTTGGGCTCTCTCAG	HSV-1 probe 1	CGTCTGGACCAACCGCCACACAGGT
hu_IL-10-fwd-1	AATAAGGTTTCTCAAGGGGCT	HSV-1 rev 1	GCAGGCACACGTAACGCACGCT
hu_IL-10-rev-1	AGAACCAAGACCCAGACATCAA	VZV-fwd 1	CGGCATGGCCCGTCTAT
hu_IL-4-fwd 1	AGAAGACTCTGTGCACCGAGTTGA	VZV-probe 1	ATTCAGCAATGGAAACACACGACGCC
hu_IL-4-rev 1	CTCTCATGATCGTCTTTAGCCTTT	VZV-rev 1	CTCGCGTGCTGCGGC
hu_IL-6F	ACCCCTGACCCAACCACAAAT	HHV-6ab fwd 1	GACAATCACATGCCTGGATAATG
hu_IL-6R	AGCTGCGCAGAATGAGATGAG	HHV-6ab rev 1	TGTAAGCGTGTGGTAATGGACTAA
hu IL-23p19 fwd 1	GAGCCTTCTCTGCTCCCTGAT	HHV-6ab-Probe 1	FAM-AGCAGCTGGCGAAAAGTGCTGTGC-TAMRA
hu IL-23p19 rev 1	AGTTGGCTGAGGCCCAGTAG	EBV-fwd-1	AAACCTCAGGACCTACGCTGC
Hu CD3e fwd	GATGCAGTCGGGCACTCACT	EBV-probe 1	TAGAGGTTTTGCTAGGGAGGAGACGTGTG
Hu CD3e rev	CAT ACCATCTTGCCCCCAA	EBV-rev-1	AGACACCGTCCTCACCAC
hu IL-12p35 fwd 1	AGCCTCCTCCTTGTCGCTACC	CMV fwd 1	GGCCGTTACTGTCTGCAGGA
hu IL-12p35 rev 1	GCCTCCACTGTGCTGGTTTTATC	CMV probe 1	CCGTATTGGTGCGCGATCTGTTCAA
Hu PD-L1 (cd274) fwd	CTTCAAGCAGGGATTCTCAACCT	CMV rev 1	GGCCTCGTAGTGAAAATTAATGGT
Hu PD-L1 (cd274) rev	TAAGTCCCACATTGCCTGCAT	HCoV229EE7	TCTGCCAAGAGTCTTGCTCG
mus IFN-alpha fwd	AGGACAGGAAGGATTTTGGA	HCoV229EE7.1	CAAAAGAACAAAAGCARGAAATCG
mus IFN-alpha rev	GCTGCTGATGGAGGTCATT	HCoVE9	AGCATAGCAGCTGTTGACGG
mus IL-12p35 fwd 1	CATCGATGAGCTGATGCAGT	HCoVE9.1	GCTCAGCAAATTGTGGATAGC
mus IL-12p35 rev 1	CAGATAGCCCATCACCCTGT	SAFV-F	CCCCCTTCAATTATAAGATTACACC
IL-1b-F	CCAAAGAAGAAGATGGAAAAGC	SAFVF2	GGACGATTGTTCTGACAACT
IL-1b-R	GGTGCTGATGTACCAGTTGGG	SAFVR	AGCTTTTCCTTTAGAGTACCTGG
16s 339F	CTCCTACGGGAGGCAGCAGT	SAFVR2	GCTAACCATTGCTTTCAAAT
16s514F	CGTGCCAGCAGCCGCGGTAAT		
16s926r	CCGTCAATTCCTTTRAGTTT		
16s806R	TCATCGTTTACGGCGTGGACTACC		

Supplementary Table 2: Mean neuropathological scores in MS and nonMS patients

	MS	nonMS	*p* value
**PGN**	3.76 ±0.49	3.33 ±0.52	2.20E-01
**LFB**	7.0 ±3.48	0.14 ±0.19	2.77E-05
**CD68**	17.9 ±3.19	8.8 ±4.03	1.11E-03
**CD3**	10.1 ±3.22	3.9 ±2.91	1.74E-03

Supplementary Table 3: Virus detection in brain

Patient group	HHV-6 A/B[1]	VZV[1]	EBV[1]	HSV-1[1]	CMV[1]	KSHV[1]	SAFV[2]	CoV[2]
**MS**	0/8	0/8	0/8	0/8	1/6	1/9	0/6	0/6
**nonMS**	3/8	0/8	1/8	0/9	3/8	0/7	0/6	1/6

1. Viral DNA detection by PCR. 2. Viral RNA detection by RT-PCR
